# Antitumor Activity of a Pyrrolobenzodiazepine Antibody–Drug Conjugate Targeting LGR5 in Preclinical Models of Neuroblastoma

**DOI:** 10.3390/pharmaceutics16070943

**Published:** 2024-07-15

**Authors:** Jianghua Tu, Yukimatsu Toh, Adela M. Aldana, Jake J. Wen, Ling Wu, Joan Jacob, Li Li, Sheng Pan, Kendra S. Carmon, Qingyun J. Liu

**Affiliations:** The Brown Foundation Institute of Molecular Medicine, Center for Translational Cancer Research, University of Texas Health Science Center at Houston, Houston, TX 77030, USA

**Keywords:** neuroblastoma, LGR5, ADC, PBD

## Abstract

Neuroblastoma (NB) is a cancer of the peripheral nervous system found in children under 15 years of age. It is the most frequently diagnosed cancer during infancy, accounting for ~12% of all cancer-related deaths in children. Leucine-rich repeat-containing G-protein-coupled receptor 5 (LGR5) is a membrane receptor that is associated with the primary tumor formation and metastasis of cancers in the gastrointestinal system. Remarkably, high levels of *LGR5* are found in NB tumor cells, and high *LGR5* expression is strongly correlated with poor survival. Antibody–drug conjugates (ADCs) are monoclonal antibodies that are covalently linked to cell-killing cytotoxins to deliver the payloads into cancer cells. We generated an ADC with an anti-LGR5 antibody and pyrrolobenzodiazepine (PBD) dimer-based payload SG3199 using a chemoenzymatic conjugation method. The resulting anti-LGR5 ADC was able to inhibit the growth of NB cells expressing LGR5 with high potency and specificity. Importantly, the ADC was able to completely inhibit the growth of NB xenograft tumors in vivo at a clinically relevant dose for the PBD class of ADCs. The findings support the potential of targeting LGR5 using the PBD class of payload for the treatment of high-risk NBs.

## 1. Introduction

Neuroblastoma (NB) is a tumor of the sympathetic nervous system that occurs in children under 15 years of age [1,2,3]. It is the most common extracranial solid tumor in childhood and the most frequently diagnosed neoplasm during infancy, accounting for ~8% of all malignancies and ~12% of all cancer-related deaths in children [1,4,5]. In approximately half of NB cases, patients are cured with minimal and sometimes no chemotherapy. The other half are the high-risk patients who are often diagnosed with metastatic disease at 18 months or older, or when their tumor exhibits the genomic amplification of the proto-oncogene *MYCN* [6,7]. High-risk patients still only have a long-term survival of ~50% despite intensive multimodal treatment that is quite toxic [6]. Recently, a monoclonal antibody (mAb) targeting the disialoganglioside GD2 (dinutuximab) became the first FDA-approved therapy for children with high-risk NB [8], but relapse still occurs in at least half of the patients [8,9]. Clearly, the discovery of novel therapeutics with a better tolerability and efficacy is still needed for children with high-risk NB.

Leucine-rich repeat-containing G-protein-coupled receptor 4, 5, and 6 (LGR4/5/6) are three related membrane receptors with a large extracellular domain (ECD) and a seven-transmembrane (7TM) domain typical of the rhodopsin family of G-protein-coupled receptors [10,11,12]. LGR4 plays critical roles in cell proliferation and migration during organ development [11,13,14,15,16,17], whereas LGR5 is specifically expressed in adult stem cells in the gastrointestinal (GI) tract and upregulated in GI cancers [18], and LGR5-positive cancer cells act like cancer stem cells that fuel the growth of tumor mass and metastasis [19,20]. R-spondins are a group of four related secreted proteins (RSPO1-4) that play essential roles in the normal development and survival of adult stem cells [21]. RSPOs function as ligands of LGR4/5/6 to potentiate Wnt signaling and support stem cell growth [22,23,24,25]. Mechanistically, RSPO-LGR4 form a complex to inhibit the function of RNF43 and ZNRF3, two E3 ligases that ubiquitinate Wnt receptors for degradation, leading to higher Wnt receptor levels and stronger signaling [26,27]. LGR5, in contrast, does not potentiate Wnt signaling through the sequestering of the E3 ligases [28]. LGR5 also regulates cell–cell adhesion, which may play an important role in cancer cell metastasis [29].

*LGR5* was first found to be one of the genes that were most enriched in NB cells selected for sphere-forming and metastasis capability [30]. Subsequent studies reported that *LGR5* expression is associated with aggressive diseases in NBs with or without *MYCN* amplification [31,32,33]. *LGR5* expression was also found to be highly enriched in end-stage tumors in the mouse model of NB driven by *MYCN* overexpression [34]. The knockdown of *LGR5* in *LGR5*-high NB cell lines led to reduced MAPK signaling and decreased cell growth [32,35]. Recently, *LGR5* expression was found to be increased in drug-resistant NB cell lines [36]. The exact roles and mechanisms of LGR5 in NB and even in GI cancers remain poorly defined.

Antibody–drug conjugates (ADCs) are monoclonal antibodies (mAbs) that are covalently linked to cell-killing cytotoxins (payloads). This approach combines the high specificity of mAbs with potent cytotoxic drugs, creating “armed” antibodies that can directly deliver the payload to antigen-enriched tumor cells while minimizing systemic toxicity [37,38,39]. The principle has now been validated by the approval of 13 ADC drugs for cancer treatment with several dozen candidates ongoing in clinical trials. Just recently, an ADC targeting the receptor kinase anaplastic lymphoma kinase (ALK) was shown to be highly effective in patient-derived ALK-high tumor models [40]. *ALK* is mutated in a small subset of high-risk NBs as a driving mechanisms and the small molecule ALK inhibitor crizotinib is being tested in children with ALK-mutated NBs [41,42]. Interestingly, *LGR*5 and *ALK* expression partially overlap in NBs. We and others have shown that anti-LGR5 ADCs are highly effective in inhibiting the growth of LGR5-positive colon cancers in xenograft models without major adverse effects [18,43]. Given the high expression of *LGR5* in high-risk NBs, we evaluated a drug conjugate of the anti-LGR5 antibody against LGR5-high NB cell lines. Here, we report that an LGR5 antibody conjugated with the PBD dimer analog SG3199 was highly effective in inhibiting the growth of LGR5-high NB cells in vitro and in vivo.

## 2. Materials and Methods

### 2.1. Cell Lines

The neuroblastoma cell lines SK-N-AS (SKNAS), SK-N-BE2 (SKNBE2), and CHP212 were purchased from the American Type Culture Collection (ATCC, Manassas, VA, USA) and cultured as suggested by ATCC. Knockout of LGR5 in the three cells lines was carried out using the vector Lenti-CRISPR2 and guide sequence CAGGAGCACACCGAGCCGGG (corresponding to nucleotide 11–30 of human LGR5 coding sequence) as described [44,45].

### 2.2. Preparation and Characterization of Antibodies and ADC

Construction of rat 8F2 into human IgG1 frame was described previously [46]. To enable transglutaminase-mediated site-specific conjugation, the Asn-297 glycosylation site of the Fc domain was mutated to Ala (N297A) using PCR-based mutagenesis and in-fusion cloning (Takara Bio Inc., San Jose, CA, USA). The coding sequences of Vh/Vl of rituximab (anti-CD20 antibody) was synthesized (Epoch Life Sciences, Missouri City, TX, USA) and converted into full-length human IgG1 using the same constant regions of 8F2. Antibody expression and production was carried out using Expi293^TM^ cells (Thermofisher, Waltham, MA, USA) and purification was carried using Protein-A affinity chromatography as described before [47].

ADC was generated using 2-step reactions: microbial transglutaminase (MTGase)-mediated site-specific conjugation and click-chemistry reaction [48,49,50,51,52]. Briefly, a solution of 8F2 with N297A mutation (10 mg/mL in PBS (pH 7.2)) underwent treatment with 8% Activa^®^ TI Transglutaminase (MTG, Ajinomoto purchased from Modernist Pantry, Portsmouth, NH, USA) and 40 molar equivalents of amino–PEG4–azide linker (BroadPharm, San Diego, CA, USA) overnight at room temperature as part of the conjugation process. The excess linker and MTG were thoroughly removed by treating the product with CaptivA^®^ Protein A Affinity Resin (Repligen, Waltham, MA, USA), followed by 2 h of vortexing at room temperature. Next, 1.5 molar equivalents of linker-drug (from a 20 mg/mL DBCO-PEG8-Val-Lys-Gly-14-aminomethy (CPT2) (Levena, San Diego, CA, USA) in dimethyl sulfoxide (DMSO) stock solution) were added to the previous step production, while keeping the residual DMSO concentration below 10% (*v*/*v*). The mixture was incubated at room temperature for 4 h, followed by purification over a size-exclusion column (Superdex 200, Hiload 16/600, Cytiva, Marlborough, MA, USA) to remove excess reagents. In this step, the resulting PDC was also buffer-exchanged into a formulation buffer (20 mM sodium succinate, 6% trehalose, and pH 5.0), and the final PDC was stored at −80 °C. Each conjugation step was monitored using LC-MS/MS system to determine linker–antibody ratio (LAD) or drug–antibody ratio (DAR). The detailed mass spectrometric analysis was as mentioned below. The other antibody–drug conjugates used in this study were prepared in the same manner.

For preparing the mass spectrometry sample, 0.25 µg of the conjugated antibodies were reduced in 10 µL antibody-reducing buffer (10 mM DTT in PBS, pH 7.2) overnight at room temperature. The mass spectrometric analysis of ADCs was carried out using a LC-MS/MS system consisting of an Agilent 6538 UHD Accurate-Mass Quadrupole Time-of-Flight (Q-TOF) coupled with an Agilent 1200 series HPLC. An Agilent PLRP-S reversed-phase column (50 × 2.1 mm, 5 μm, 1000 Å) was used for LC separation. The solvent system consisted of buffer A—2% acetonitrile, 97.9% water, and 0.1% formic acid, and buffer B—80% acetonitrile, 19.9% water, and 0.1% formic acid. A gradient of 25–90% B was applied over 20 min at a flow rate of 0.2 mL/min. The samples were injected after being diluted with 25% acetonitrile, 74.9% water, and 0.1% formic acid. The Q-TOF was operated in ESI positive mode with capillary voltage of 3500 V, drying gas flow rate of 7 L/min, nebulizer at 35 psi, and the source temperature of 325 °C. Spectra were acquired in MS1 scan from 4.5–20 min over the mass range of 650–2800 *m*/*z*. The raw data were processed using Agilent MassHunter BioConfirm software (Version B.04.00), which uses the Maximum Entropy deconvolution algorithm for the accurate molecular mass calculation. The deconvoluted mass range was set at 20,000 to 100,000 Daltons. According to the formula LAR/DAR = 2 × (Σ weighted peak area of heavy chain (= linker/drug load × weighted peak area %)/100 (ref)), the LAR/DAR of conjugated antibody was calculated.

### 2.3. Immunofluorescence and Immunoblotting (Western Blot)

For LGR5 internalization, NB cells including SKNAS, SKNBE2, CHP212, and CHP212-LGR5KO cells were seeded into 8-well chamber slides and cultured overnight. The following day, cells were incubated with 8F2 at 10 μg/mL for one hour at 37 °C/5% CO_2_ in complete culture media to enable binding and internalization. After washing the cells with PBS, they were fixed in 4.2% for paraformaldehyde and permeabilized in 0.1% saponin before being incubated with goat anti-human-Alexa-488 for one hour at room temperature. Nuclei were counterstained with TO-PRO-3, and the images were acquired using a confocal Leica TCS SP5 microscope with LAS AF Lite software 2.7.7.7. Immunoblotting of LGR5 was carried out using the LGR5 antibody (ab219107, Abcam, Waltham, MA, USA) under standard conditions.

### 2.4. In Vitro Binding Analysis and Cytotoxic Assay

Binding affinity of ADCs to LGR5 were determined using HEK293 cells over-expressing human LGR5 as described in detail before [22,43]. Kd was calculated using GraphPad Prism 8 software. For cytotoxicity assays, cells (3 × 10^3^/well) were seeded in 96-well plates (Costar Assay Plate, Corning, Corning, NY, USA) and serial dilutions of ADCs (0–10 nM) were added. The cells were incubated for 5 days and cell viability was measured by CellTiter-Glo^®^ Luminescent Cell Viability Assay (Promega, Madison WI, USA) using TECAN infinite^®^ M1000 (Tecan Austria GmbH, Grödig, Austria). IC_50_ was calculated using GraphPad Prism 8 software (GraphPad Prism, Boston MA, USA).

### 2.5. In Vivo Studies

Animal studies were carried out in strict accordance with the recommendations of the Institutional Animal Care and Use Committee of the University of Texas at Houston (Protocol number AWC-21-0094). For SKNAS xenograft study, female 9-week-old nu/nu mice (Charles River Laboratories) were subcutaneously inoculated with 5 × 10^6^ cells in 1:1 mixture with Matrigel (BD Biosciences, Franklin Lakes, NJ, USA). After 2 weeks, when tumor size reached approximately ~100 mm^3^, mice were randomized into 4 groups at 5 mice per group and given vehicle (PBS), 8F2-SG3199 or R20-SG3199, at the indicated dose levels once per week by intraperitoneal injection for a total of five doses. Mice were routinely monitored for morbidity and mortality. Tumor volumes were measured bi-weekly and estimated by the formula: tumor volume = (length × width^2^)/2.

### 2.6. Statistical Analysis

All data were analyses using GraphPad Prism 8 software. Data are expressed as mean ± SEM or SD as indicated in the Results section. The expression and survival data of the TCGA Target cohort of neuroblastoma were analyzed using Kaplan–Meier analysis with log-rank (Mantel–Cox) test for *p*-value calculation. In vitro experiment was determined using one-way analysis of variance (ANOVA) followed by Tukey’s multiple comparison test, or Student’s unpaired two-tailed *t*-test for comparisons between two groups. For tumor volume analysis, one-way ANOVA with Dunnett’s multiple comparison test was employed. Survival data were analyzed using Kaplan–Meier analysis with log-rank (Mantel–Cox) test for *p*-value calculation. *p* ≤ 0.05 was considered statistically significant.

## 3. Results

### 3.1. LGR4 and LGR5 Are Expressed in NBs and High LGR5 Expression Is Associated with Poor Survival

**Figure 1:**
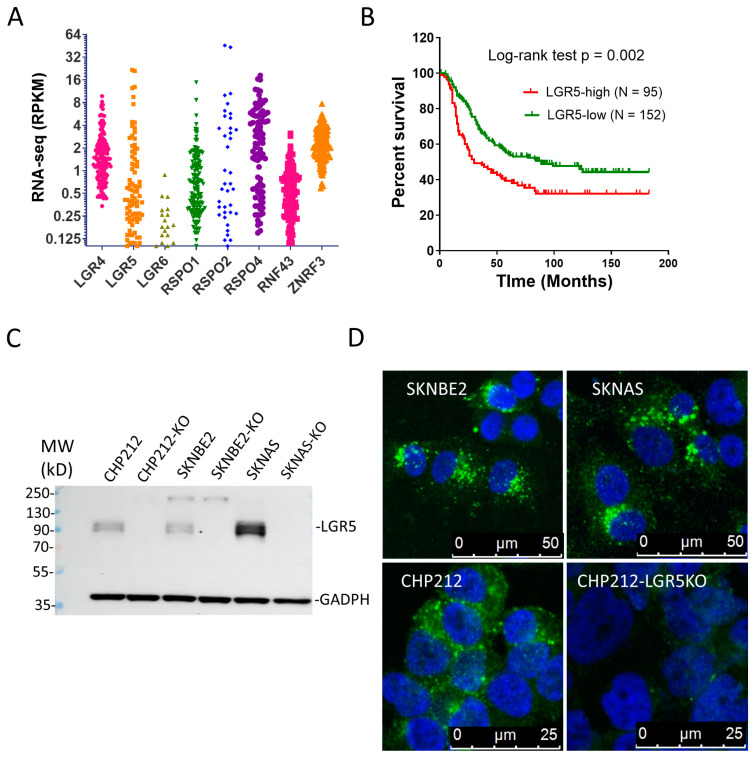
Expression profile of RSPO-LGR-RNF43/ZNRF in NBs and analysis of LGR5 expression in cell lines. (**A**) TCGA’s RNA-seq data of RSPO1/2/4, LGR4/5/6, and RNF43/ZNRF3 in the NB Target cohort. (**B**) Kaplan–Meier analysis of LGR5 expression vs. survival in the TCGA Target cohort. (**C**) Western blot result of LGR5 in three NB cells lines and their LGR5-knockout derivatives. Glyceraldehyde-3-phosphate dehydrogenase (GAPDH) is loading control. (**D**) Internalization of 8F2 bound to LGR5 by confocal microscopy. Green vesicles represent internalized LGR5. Blue = nuclei.

We analyzed the RNA-seq data of The Cancer Genome Atlas (TCGA) NB cohort (Target, 2018) for expression of LGR4/5/6, RSPO1/2/4 (no data for RSPO3), and RNF43/ZNRF3. Both LGR4 and LGR5 were expressed in a large subset of tumors with LGR5 having a broader distribution (Figure 1A). Little expression of LGR6 was observed. RSPO2 and RSPO4 were expressed in approximately half of the tumors at moderate to high levels and ZNRF3 was found at higher levels than RNF43 in most of the tumors (Figure 1A). These data suggest the LGR-RSPO-ZNRF3/RNF43 were expressed in approximately half of the tumors at moderate to high levels. We then analyzed the relationship between expression and survival and found that a high LGR5 expression was strongly correlated with a worse clinical outcome (median survival of 30 vs. 89 days, log-rank test *p* = 0.002, Figure 1B), consistent with previous findings [32]. Next, we analyzed RNA-seq data of 24 NB cell lines in the Cancer Cell Line Encyclopedia (CCLE) database and found that LGR5 expression was relatively high in about half of the cell lines whereas LGR4 was expressed at lower levels in nearly all the cell lines (Supplementary Table S1). In contrast, little expression of LGR6 was detected (Supplementary Table S1). Interestingly, little to no expression of RSPO1 and RSPO2 were found in these cell lines (Supplementary Table S1), suggesting that their expression in primary tumors was most likely from stromal or other non-tumor cells. One cell line (KPNSI9S) has a high expression of RSPO3 while six cell lines express RSPO4 at moderate levels, suggesting that they were expressed in tumor cells in the primary tumors. Low levels of ZNRF3 were found in nearly all the cell lines while no expression of RNF43 was observed (Supplementary Table S1), consistent with the relatively high expression of ZNRF3 in primary tumors (Figure 1A).

We selected three NB cell lines with a high expression of LGR5 (SKNBE2, SKNAS, and CHP212) and confirmed the expression by immunoblotting with anti-LGR5 antibody (Figure 1C). LGR5 was then knocked out in each of the three cell lines using the CRISPR/Cas9 method, and the loss of expression was confirmed (Figure 1C). Using the LGR5 antibody, 8F2, which was shown to be able to bind to native LGR5 [43], we found that 8F2 was quickly internalized in the three cell lines after incubating for one hour at 37 °C (Figure 1D). Importantly, CHP212 cells with KO of LGR5 showed the loss of 8F2 binding and internalization (Figure 1D, lower right panel). Overall, these results confirmed that LGR5 was expressed in nearly half of primary tumors and cell lines at high levels and a high expression of LGR5 was associated with poor survival. Importantly, the LGR5-bound antibody underwent rapid internalization.

### 3.2. Conjugation of SG3199 to Anti-LGR5 Antibody Using Chemoenzymatic Method

**Figure 2:**
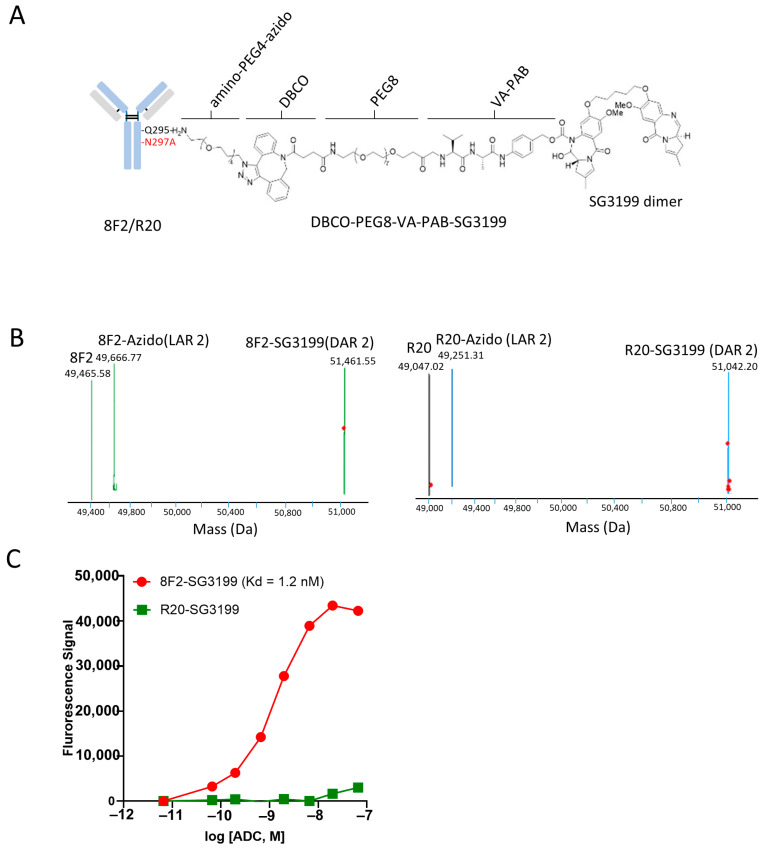
Generation of anti-LGR5 ADC and its binding to LGR5-expressing cells. (**A**) diagram of LGR5 antibody 8F2 or control antibody R20 conjugated with PEG4-azido-DBCO-PEG8-VA-PAB-SG3199. (**B**) Mass spectra of 8F2-SG3199 and R20-SG3199. (**C**) Saturation binding analysis of 8F2-SG3199 and R20-SG3199 on HEK293T cells stably expressing LGR5. Kd values were then calculated using GraphPad Prism 8 software. All assays were performed in triplicate.

Given the high expression of LGR5 in NB cells, we reasoned that anti-LGR5 ADC may be able to inhibit the growth of NB cells. Previously, we generated anti-LGR5 ADC using the 8F2 antibody and the auristatin-based linker-payload mc-VC-PAB-MMAE (monomethyl auristatin E) by cysteine-linked chemical conjugation [43]. As microtubule inhibitors such as MMAE may have a limited efficacy in solid tumors, we set out to test the new generation of pyrrolobenzodiazepine dimer SG3199 which acts as a highly potent cytotoxin by cross-linking DNA [53]. Conjugation was achieved by a site-specific method using a two-step chemo-enzymatic reaction with microbial transglutaminase and click chemistry [49]. An N297A mutation was introduced into the Fc domain of 8F2, which abolishes antibody glycosylation and renders Q295 a recipient residue for the microbial transglutaminase [49]. Amino–PEG4–azide was first conjugated to the Q295 of the antibody, followed by strain-promoted alkyne–azide cycloaddition (SPACC) reaction using Dibenzocyclooctyne (DBCO)-PEG8-VA-PAB-SG3199 (Figure 2A) [50,54]. Each step was verified by mass spectrometry (Figure 2B). We also generated a non-targeting, control ADC using the anti-CD20 antibody rituximab (designated R20) using the same linker-payload and conjugation method (Figure 2A,B).

The resulting ADCs, termed 8F2-SG3199 and R20-SG3199, were tested for binding to LGR5. As shown in Figure 2C, 8F2-SG3199 showed potent binding to HEK293T cells expressing LGR5 with a Kd of 1.2 nM, similar to the affinity of unconjugated 8F2 [43]. R20-SG3199, in contrast, showed minimal binding to LGR5 (Figure 2C).

### 3.3. 8F2-SG3199 Displayed Highly Potent Cytotoxic Activity in LGR5-High NB Cells

**Figure 3:**
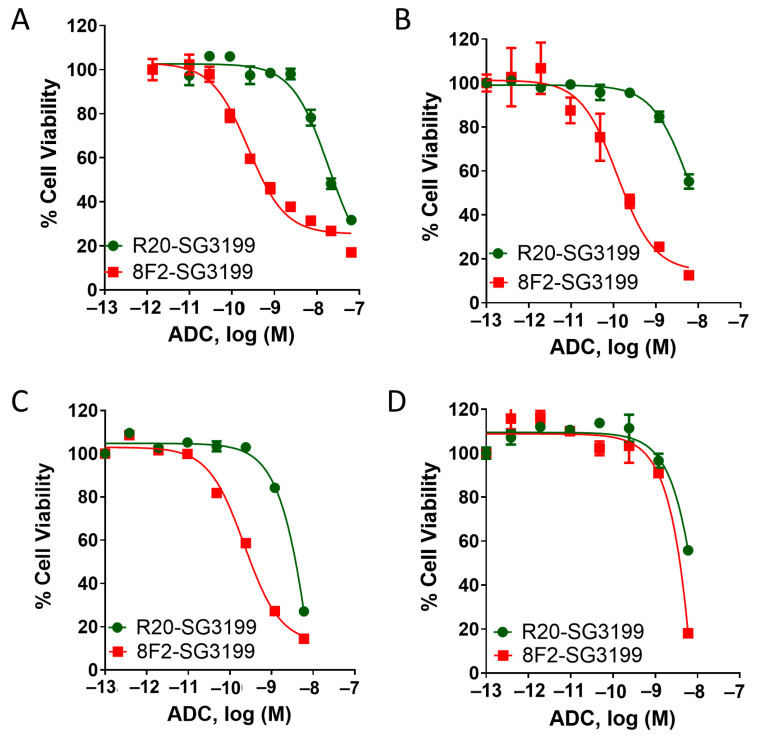
Analysis of cytotoxic activity of 8F2-SG3199 and R20-SG3199 in SKNBE2, SKNAS, and CHP212 NB cell lines and CHP212-LGR5KO cells: (**A**) SKNAS, (**B**) SKNBE2, (**C**) CHP212, and (**D**) CHP212-LGR5KO cell.

We then evaluated the cytotoxic potency and efficacy of the 8F2-SG3199 and R20-SG3199 in three NB cells lines (SKNAS, SKNBE2, and CHP212) expressing LGR5 at high levels and CHP212-LGR5KO cells (Figure 1C,D). In all three cell lines, 8F2-SG3199 displayed IC50s of 0.1 to 0.2 nM with the near complete inhibition of cell growth whereas R20-SG3199 was ~100× less potent (Figure 3A–C). The cytotoxic activity of control ADC is most likely due to the non-specific uptake by the cells or non-specific binding of R20-SG3199 at high concentrations. In CHP212-LGR5KO cells, 8F2-SG3199 and R20-SG3199 had a similar potency (Figure 3D). These results indicated that 8F2-SG3199 had a potent and specific cytotoxicity that was mediated by LGR5.

### 3.4. 8F2-SG3199 Inhibited Tumor Growth In Vivo

**Figure 4:**
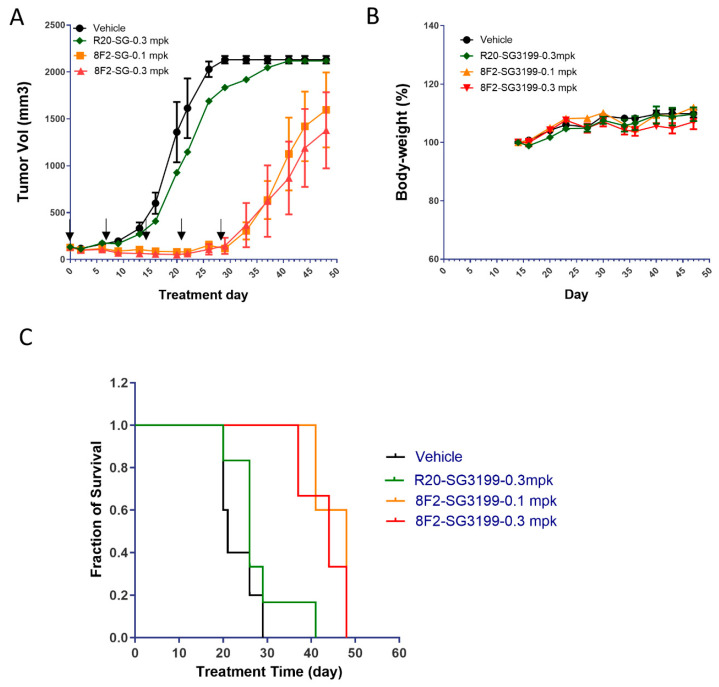
Anti-tumor potency and efficacy of 8F2-SG3199 in xenograft model of SKNAS cells. (**A**) Growth curves of tumors treated with vehicle, 8F2-SG3199, or R20-SG3199 at the indicated dose levels. Days of the five injections were marked by arrows. (**B**) Body weight curves. (**C**) Kaplan–Meier survival plot.

To determine if 8F2-SG3199 was able to inhibit tumor growth in vivo, we tested the ADC in xenograft models of SKNAS with R20-SG3199 as control. SKNAS cells were implanted subcutaneously, and, when tumors reached ~100 mm^3^, 8F2-SG3199 was given at 0.1 mg/kg and 0.3 mg/kg, whereas R20-SG3199 was given at 0.3 mg/kg, once per week for a total of five doses (Figure 4A). 8F2-SG3199 was able to inhibit tumor growth completely at both dose levels, whereas the control ADC R20-SG3199 had no significant effect at 0.3 mg/kg when compared to the vehicle group (Figure 4A). Neither gross toxicity nor significant loss in body weight was noticed in any of the groups (Figure 4B). After the treatment with 8F2-SG3199 stopped, the tumors started to grow and eventually reached the size for the termination of the study (Figure 4A and Supplementary Figure S2). Nevertheless, 8F2-SG3199 treatment at either 0.1 mg/kg or 0.3 mg/kg led to a significant increase in survival (log-rank test *p* = 0.01, Figure 4C). The median survival of the 8F2-SG3199-treated animal was 48 days (0.1 mg/kg) and 44 days (0.3 mg/kg), whereas those of the control groups were 21 days (vehicle) or 26 days (R20-SG3199 at 0.3 mg/kg). Overall, these results suggest that 8F2-SG3199 was able to inhibit tumor growth but could not eradiate tumors. Further studies are required to understand the potential mechanisms of drug resistance.

## 4. Discussion

ADCs have emerged as a major modality of targeted cancer treatment with a large number of drug candidates approved or in the late stages of development [55,56,57]. Anti-LGR5 ADCs have been shown to be effective in preclinical models of colon cancer [18,43]. Here, we show that LGR5 is highly expressed in a subset of NB tumors and cell lines and that anti-LGR5 ADC with a PBD payload is highly effective in inhibiting the growth NB cells with a high LGR5 expression in vitro and in vivo.

LGR5 is best known as a marker of adult stem cells in the gastrointestinal tract, as well as in the skin and some other tissues [58]. It is also known to be increased in expression and associated with cancer cell stemness in cancers of the gastrointestinal system [17,18,43]. The high expression of LGR5 in high-risk neuroblastoma is surprising as LGR5 is typically found in epithelial cells and fibroblasts [58,59,60]. Single-cell sequencing data of the brain showed that LGR5 is expressed at low levels in neurons with a much higher expression in oligodendrocytes [60]. The high expression of LGR5 in NB cells may be due to the elevated Wnt signaling in NB since LGR5 is a Wnt target gene [58,61]. The function of LGR5 in NB cells remains poorly understood. Previously, it was reported that the knockdown of LGR5 by siRNA led to decreased MAP kinase signaling and apoptosis [32]. We generated stable LGR5 knockout cells in three NB cells lines (SKNAS, SKNBE2, and CHP212) using the CRIPR/cas9 method and found no consistent difference in cell growth between parental and knockout cells. The effect of the LGR5 knockout on tumor formation and growth in vivo remains to be determined.

Given the high expression of LGR5 in NB tumor cells, we tested the potential of ADCs targeting LGR5 for the treatment of high-risk NB using the PBD class of the payload SG3199. The camptothecin derivative topotecan is used as part of standard chemotherapy in the treatment of high-risk NB [1,9]. The choice of SG3199 was for the potential of using the ADC together with standard chemotherapy. SG3199 is used in one FDA-approved ADC (loncastuximab tesirine) [53,62]. The resulting ADC (8F2-SG3199) showed a potent and specific cytotoxicity in NB cells expressing high levels of LGR5 in vitro (Figure 3). To evaluate its effect in vivo, 8F2-SG3199 was dosed at 0.1 mg/kg and 0.3 mg/kg in a xenograft model of SKNAS cells. Both dose levels were able to completely inhibit tumor growth but could not eradicate tumors (Figure 4A). SG3199-based ADCs were only tolerated at relatively low dose levels. Loncastuximab tesirine (anti-CD19 ADC with SG3199 as the payload) was used in the clinic at an initial dose of 0.15 mg/kg followed by 0.075 mg/kg every three weeks [63]. Our finding that 8F2-SG3199 was effective and well-tolerated at 0.1 mg/kg in mouse strongly support its potential as a treatment for high-risk NB. Additional studies, including testing at lower dose levels and more xenograft models, as well as detailed toxicology studies, are still required to further support the use of SG3199-based ADCs for NB treatment. Of note, 8F2 is specific for human LGR5 [46], and, thus, the potential target-mediated toxicity could not be assessed with 8F2-SG3199 in the mouse. However, the targeted-mediated toxicity of ADCs is relatively rare and most toxicities are due to the non-specific uptake of ADC or release of the payload [64,65]. Overall, our data strongly support the potential of anti-LGR5 ADC using SG3199 as the payload for the treatment of high-risk NBs.

## 5. Conclusions

In conclusion, we confirmed that LGR5 is expressed in a subset of NBs and a high expression is strongly associated with poor survival. Anti-LGR5 ADC with a potent payload was able to inhibit the growth of NB cells expressing high levels of LGR5 with a high potency and specificity. Importantly, the ADC was able to inhibit tumor growth in vivo at a clinically relevant dose. Further studies are required to understand the roles and functions of LGR5 in NB and the mechanism of drug resistance to the ADC.

## Data Availability

The data presented in this study are available in this article.

## Supplementary Materials

The following supporting information can be downloaded at: https://www.mdpi.com/article/10.3390/pharmaceutics16070943/s1, Figure S1: Mass spectra of 8F2-SG3199 and R20-SG3199; Figure S2: Growth curves of individual mice in Figure 4A; Table S1: RNA-seq date (RPKM) of RSPO-LGR-RNF43/ZNRF3 of NB cell lines characterized by CCLE.
