# Supplementary material for: Antitumor Activity of a Pyrrolobenzodiazepine Antibody–Drug Conjugate Targeting LGR5 in Preclinical Models of Neuroblastoma
Source: Pharmaceutics. 2024 Jul 15;16(7):943. doi: 10.3390/pharmaceutics16070943 (PMC11279891; doi:10.3390/pharmaceutics16070943)
Supplement: Supplementary file 1 [file pharmaceutics-16-00943-s001.zip › pharmaceutics-3092489-supplementary.pdf]

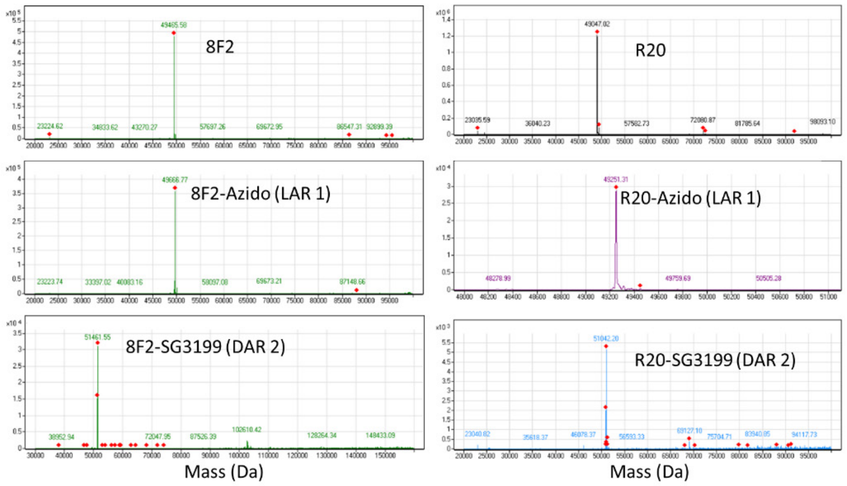

Supplementary Figure S1. Mass spectra of 8F2-SG3199 and R20-SG3199

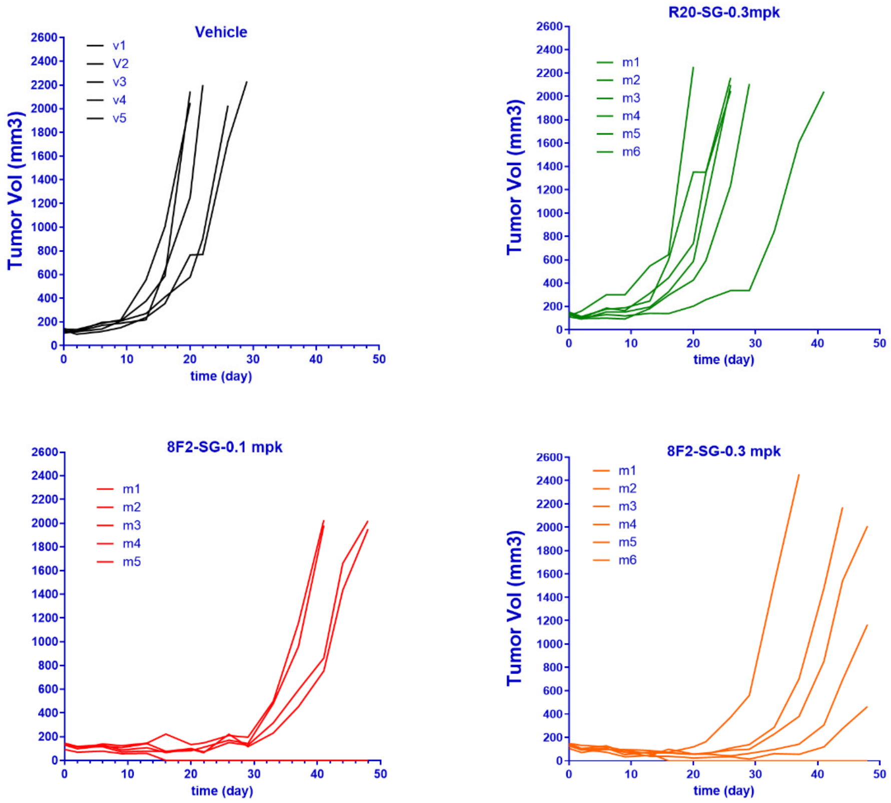

Supplementary Figure S2. Growth curves of individual mice in Figure 4A.

Supplementgary Table S1. RNA-seq data (RPKM) of RSPO-LGR-RNF43/ZNRF3 of NB cell lines characterized by CCLE									
cell line	Gene								
	LGR4	*LGR5*	*LGR6*	*RSPO1*	*RSPO2*	*RSPO3*	*RSPO4*	*RNF43*	*ZNRF3*
CHLA15	2.6	115.7	0.0	0.2	0.0	0.0	0.8	0.0	3.3
CHP126	2.5	1.6	0.0	0.0	0.2	0.0	0.1	0.0	4.8
CHP212	0.9	30.1	0.0	0.0	0.0	0.1	0.0	0.0	4.3
COGN278	6.7	30.7	0.0	0.1	0.2	0.0	3.4	0.0	4.5
COGN305	10.8	0.1	0.0	0.1	0.1	0.0	4.8	0.0	3.7
GIMEN	2.8	5.4	0.0	0.0	0.0	1.2	0.0	0.0	2.0
IMR32	4.3	0.7	0.0	0.0	0.0	0.0	0.0	0.0	22.1
KELLY	3.1	3.0	0.0	1.6	0.0	0.0	0.1	0.0	6.7
KPNRTBM1	6.4	17.9	0.0	0.1	0.0	0.0	0.7	0.0	6.0
KPNSI9S	9.2	7.7	0.0	0.0	0.1	20.3	6.0	0.1	3.8
KPNYN	5.0	30.0	0.0	0.0	0.0	0.0	0.0	0.0	9.1
LAN2	7.3	5.3	0.0	0.1	0.9	0.0	8.8	0.5	2.7
MHHNB11	5.3	4.0	0.0	0.0	0.0	0.0	4.8	0.0	8.8
NB1	2.6	16.0	0.0	0.0	0.1	0.0	0.0	0.0	9.0
NB1643	3.8	15.0	0.0	0.0	0.0	0.0	0.0	0.0	4.0
NH6	3.1	19.5	0.0	0.0	0.2	0.0	0.0	0.0	9.7
NMB	2.7	0.7	0.0	0.5	0.1	0.0	0.0	0.0	4.0
SIMA	4.2	6.4	0.1	0.4	0.0	0.0	0.1	0.0	5.7
SKNAS	1.5	42.3	0.0	0.0	0.2	0.0	13.2	0.0	4.0
SKNBE2	5.3	53.9	0.0	0.0	0.0	0.0	1.8	0.0	6.3
SKNDZ	7.8	0.5	0.0	0.0	0.1	0.0	0.1	0.0	6.4
SKNFI	4.6	0.7	0.0	0.0	0.0	0.0	0.3	0.0	4.2
SKNSH	3.3	15.7	0.0	0.0	0.0	0.0	0.6	0.0	5.1
Y79	3.1	0.0	1.2	0.8	0.0	0.0	0.0	0.1	3.5
